# Tolvaptan Treatment in Children with Chronic Hyponatremia due to Inappropriate Antidiuretic Hormone Secretion: A Report of Three Cases

**DOI:** 10.4274/jcrpe.4531

**Published:** 2017-09-01

**Authors:** Gerdi Tuli, Daniele Tessaris, Silvia Einaudi, Luisa De Sanctis, Patrizia Matarazzo

**Affiliations:** 1 University of Turin, Regina Margherita Children’s Hospital, Department of Public Health and Pediatrics, Division of Pediatric Endocrinology, Turin, Italy

**Keywords:** Tolvaptan, children, hyponatremia, syndrome of inappropriate antidiuretic hormone secretion

## Abstract

Hyponatremia is the most common electrolyte disorder among hospitalized patients and it is sometimes considered as a poor outcome predictor. Its correction is thus indicated, even in asymptomatic patients. The conventional treatment consists of fluid restriction in presence of euvolemia or hypervolemia; loop diuretics are used in some hypervolemic conditions such as cardiac heart failure, liver cirrhosis and nephrotic syndrome, while intravenous isotonic or hypertonic solutions are administered in hypovolemic conditions. The utilization of demeclocycline and urea is not indicated in pediatric ages due to lack of data on their toxicity and poor tolerance. Recently, a new therapeutic option has been developed, a class of non-peptide arginine vasopressin receptor antagonists called vaptans. Tolvaptan is the only such agent approved in Europe for the treatment of hyponatremia caused by syndrome of inappropriate antidiuretic hormone secretion (SIADH) in adults. In USA, tolvaptan and conivaptan have been approved for treatment of euvolemic and hypervolemic hyponatremia. Few data are so far available in paediatric patients, since only one trial has been registered in Europe which includes children and adolescents, but this trial is still ongoing. Here, we report three children with chronic hyponatremia due to SIADH in which tolvaptan has been used successfully.

**What is already known on this topic?**

Actually, the European Medicines Agency has only approved tolvaptan, a selective V2-receptor antagonist, for the treatment of hyponatremia due to syndrome of inappropriate antidiuretic hormone secretion (SIADH) in adults, whereas the United State Food and Drug Administration recommend tolvaptan and conivaptan for the treatment of both euvolemic and hypervolemic hyponatremia in adults. Many researchers have reported their results with tolvaptan treatment in hypervolemic hyponatremia due to heart failure or polycystic kidney disease. Tolvaptan has been used successfully in two infants to treat hyponatremia due to SIADH. However, treatment with vaptan in paediatric age groups has not been licensed yet neither in Europe nor in the USA.

**What this study adds?**

In this paper, we report the use of tolvaptan in 3 children affected by chronic euvolemic hyponatremia due to SIADH. Tolvaptan has been used respectively for 4 and 3 years in the first two patients and for 3 months in the last patient. To date, this is the longest period of drug utilization in children with euvolemic hyponatremia.

## INTRODUCTION

Hyponatremia is defined as a serum sodium level below 135 mmol/L and represents the most frequent electrolyte disorder among hospitalized patients (1,2). Hyponatremia can be classified on the basis of volemic state, i.e. hypovolemic, euvolemic, and hypervolemic, or on the basis of degree of severity of the salt and/or water wasting resulting from different pathogenetic disorders. The symptoms related to this extremely heterogeneous condition depend on many factors and may vary from none to cerebral oedema, seizures, and coma. Particular caution is required in its treatment to avoid rapid correction and consequent osmotic demyelination.

Hypovolemic hyponatremia is a frequent condition in paediatric patients and isotonic or hypertonic saline solution is still the mainstay of treatment (1,2,3,4,5,6,7). In older children, hypervolemic hyponatremia usually follows cardiac heart failure, liver cirrhosis, and nephrotic syndrome. Treatment usually consists of fluid restriction together with treatment of the underlying disease. Loop diuretics are also available for treatment in children, while demeclocycline and urea are not allowed due to lack of adequate data on their toxicity and tolerance.

Euvolemic hyponatremia is a typical feature of inappropriate secretion of antidiuretic hormone (SIADH). In children, it is usually associated to hypothalamic-chiasmatic tumours, meningitis-encephalitis, and sepsis. An autonomous arginine-vasopressin (AVP) secretion, independent from plasma osmolality or from the volemic state, is present. AVP is the physiological hypothalamic hormone that regulates osmolality by controlling urinary volume and composition. Normally, it is secreted in response to increases in plasma tonicity or to decreases in plasma volume and activates three types of receptors. In euvolemic hyponatremia, the gold standard treatment is fluid restriction only (2,3), but this approach is often ineffective or difficult to achieve as these patients also have a lowered osmotic threshold for thirst. Compliance is thus really poor and frequently use of isotonic or hypertonic saline solution is needed.

An alternative treatment is actually represented by non-peptide arginine-vasopressin-receptor antagonists, named vaptans (8,9,10). The blocked pathway of AVP signaling inhibits water resorption and results in excretion of diluted urine or “aquaresis” (10). The agents can be used in euvolemic and hypervolemic hyponatremias but are contraindicated in hypovolemic states. Several trials have proven the effectiveness of vaptans in increasing serum sodium levels. As the vaptans-dependent urine generation is electrolyte free, the utilization of these agents makes repletion of electrolytes unnecessary (6).

Actually, the European Medicines Agency (EMA) has only approved tolvaptan, a selective V2-receptor antagonist, for the treatment of hyponatremia due to SIADH, whereas the US Food and Drug Administration (FDA) has approved both tolvaptan and conivaptan as non-selective V1/V2 receptor antagonists for the treatment of both euvolemic and hypervolemic hyponatremia. Their dosage varies from 15 to 60 mg daily or 0.1 to 0.8 mg/kg (9,10). To date, vaptans are not in use in the treatment of acute hyponatremia.

Few data are available on use of vaptans in the pediatric age group (11,12,13,14,15,16,17,18). To date, there is only one ongoing trial registered in Europe which includes children and adolescents. Thus, the use of vaptans in children is not yet included in standard treatment schedules.

We report three pediatric cases of chronic severe hyponatremia due to SIADH who received tolvaptan treatment once the hyponatremia became symptomatic.

## CASE REPORTS

### Case 1

**Figure 1:**
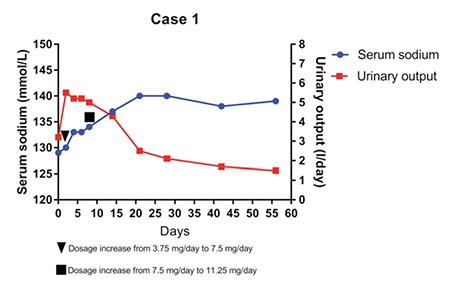
Serum sodium and daily urinary output at initiation of tolvaptan treatment and trend in the first two months of treatment

This male patient was a case of ROHHAD syndrome (rapid-onset obesity with hypothalamic dysfunction, hypoventilation, and autonomic dysregulation) referred for endocrinological follow-up. The initial hormonal evaluations at age 5 years had revealed central hypothyroidism requiring treatment with L-thyroxine and then central adrenal insufficiency at age 6 years, at which time, treatment with cortone acetate was started. Over the years of endocrinological follow-up, he presented with usually asymptomatic hypernatremia alternating with hyponatremia due to hypothalamic antidiuretic hormone (ADH) release dysregulation. At age of 7 years, a grand mal seizure episode occurred; serum sodium level was decreased at 125 mmol/L. The acute episode was treated with intravenous phenobarbital and isotonic saline solution. Brain magnetic resonance imaging (MRI) and computed tomography (CT) scan did not show any relevant abnormality, while electroencephalogram showed nonspecific alterations which were attributed to hyponatremia. Evaluation of the clinical (body weight stable at 56 kg, arterial blood pressure 110/70 mmHg, urinary output 1500 mL/24 h) and laboratory findings (plasma sodium 134 mmol/L (reference range 135-145), plasma osmolality 268 mOsm/kg, urea 33 mg/dL (reference range 20-80), creatinine 0.34 mg/dL (reference range 0.25-0.85), copeptin 14 pmol/L (reference range 3-8), urine sodium 96 mmol/L, urine osmolality 484 mOsm/kg) led to a diagnosis of SIADH. Upon cessation of intravenous administration of the isotonic and hypertonic saline solution, the serum sodium level remained unstable ranging from 127 to 133 mmol/L. For this reason, we decided to start an oral low-dose treatment with tolvaptan at 3.75 mg (0.06 mg/kg/day), which was increased to 7.5 mg and then to 11.25 mg after few days (Figure 1).

At present, after 4 years of tolvaptan treatment (present dose 11.25 mg/day, i.e. 0.2 mg/kg/day), the serum sodium levels are stable (ranging from 137 to 144 mmol/L) and no acute nor severe symptoms due to hyponatremia have been observed.

### Case 2

A 4-year-old girl with a large sellar and suprasellar tumour developed chronic euvolemic hyponatremia due to SIADH. The diagnosis was established after brain MRI and CT scan were performed for neuro-psychomotor development delay, visual loss and hyponatremia. The neurosurgical biopsy revealed a low grade ganglioglioma, so neither chemotherapy nor radiotherapy were proposed. Once the diagnosis was made, regular endocrinological and oncological follow-up was established.

At diagnosis, the patient had asymptomatic hyponatremia (serum sodium ranging from 127 to 131 mmol/L), central precocious puberty, for which a treatment with LHRH-analogue was begun, and severe hypothalamic obesity.

At age 8 years, she had a grand mal seizure episode. Serum sodium level at admission to the Emergency Department was 122 mmol/L. Brain MRI and CT scan did not show any increase of the tumour mass size. Electroencephalogram showed non-specific metabolic wave alterations, thus the pathogenesis of the grand mal seizure was attributed to hyponatremia. Pituitary-thyroid and pituitary-adrenal axis functionality was normal. SIADH was confirmed by clinical and laboratory findings (body weight stable at 35 kg, arterial blood pressure 115/80 mmHg, urinary output 1300 mL/24 h, plasma sodium 133 mmol/L (reference range 135-145), plasma osmolality 265 mOsm/kg, urea 30 mg/dL (reference range 20-80), creatinine 0.30 mg/dL (reference range 0.25-0.85), copeptin 16.6 pmol/L (reference range 3-8), urine sodium 144 mmol/L, urine osmolality 502 mOsm/kg).

**Figure 2:**
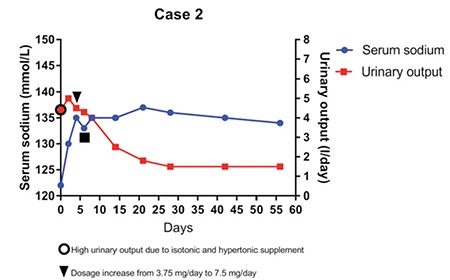
Trend of serum sodium and daily urinary output in the first two months after tolvaptan treatment initiation

The acute episode was treated with intravenous phenobarbital and hypertonic saline solution and with orally administered levetiracetam at discharge. As chronic hyponatremia became symptomatic over time, tolvaptan was started in a dose of 3.75 mg/day (0.1 mg/kg/day, weight 35 kg), then increased to 7.5 mg (Figure 2).

No other seizure episode was observed and levetiracetam withdrawal was decided after 8 months. Actually, after 3 years of treatment, serum sodium levels are nearly normal ranging from 133 to 137 mmol/L and the tolvaptan dosage is 11.25 mg/day (0.32 mg/kg/day). No severe hyponatremia has been registered despite the increase in the size of the tumour mass and central adrenal insufficiency onset.

### Case 3

**Figure 3:**
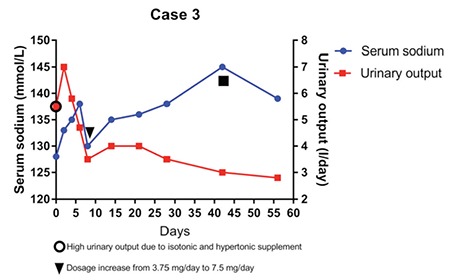
Trend of serum sodium and daily urinary output in the first months after tolvaptan treatment initiation and withdrawal timing of associated hyponatremia treatments

This 5-year-old boy developed chronic euvolemic hyponatremia due to SIADH after neurosurgical partial removal of a hypothalamic-chiasmatic astrocytoma. Post-surgical chemotherapy regimen treatment was started and an endocrinological follow-up was established to evaluate hypothalamic-pituitary axis functionality. At this time, he had asymptomatic hyponatremia with serum sodium levels ranging from 123 mmol/L to 130 mmol/L. As he developed central hypothyroidism and secondary adrenal insufficiency, a substitutive treatment for these conditions was started. He also developed central precocious puberty for which a LHRH-analogue treatment was started. At age 8 years, after the beginning of a second chemotherapy regimen treatment due to tumour size increase, hyponatremia became symptomatic with headache, nausea, asthenia, and seizures which persisted after chemotherapy withdrawal. Corticosteroid dosage increase (oral cortone acetate 40-50 mg/m^2^/day), mineralocorticoid treatment (oral fludrocortisone, 0.1 mg/day), and salt supplement (oral NaCl 3 g/day) were not sufficient to maintain serum sodium at acceptable levels, thus we started low-dose tolvaptan treatment (3.75 mg/day, 0.05 mg/kg/day, weight 83 kg) with prompt normalization of serum sodium (136-141 mmol/L) as shown in Figure 3.

After 3 months, the tolvaptan dosage has been increased to 7.5 mg (0.09 mg/kg/day), the corticosteroid dosage was reduced to a substitutive range (10-15 mg/m^2^/day), the mineralocorticoid treatment and the supplemented salt were discontinued, with serum sodium levels remaining stable at 135-144 mmol/L.

## DISCUSSION

A hypothalamic-pituitary dysregulation of the ADH secretion is the most frequent cause of SIADH in pediatric ages, whereas in adults, SIADH is mostly associated with paraneoplastic conditions.

SIADH can be clinically indistinguishable from the nephrogenic syndrome of inappropriate antidiuresis (NSIAD) which is an inherited form of renal water retention. Family history and suppressed ADH levels which were absent in our patients can help orientation towards this diagnosis. Moreover, patients with NSIAD do not respond to AVPR2 antagonists. Gold standard treatment of SIADH is fluid restriction which is difficult to achieve in pediatric ages. This approach is often ineffective as patients affected by SIADH have also a lower osmotic threshold for thirst.

Here, we report the results of tolvaptan treatment in three pediatric patients who developed symptomatic chronic hyponatremia due to SIADH. Other causes of euvolemic hyponatremia such as hypothyroidism or hypocortisolism were excluded in case 2. The patients described in cases 1 and 3 were already taking substitutive treatment for both conditions when they developed severe neurologic symptoms due to hyponatremia. The decision for using tolvaptan was taken due to the severity of symptoms related to the hyponatremic condition and presence of a chronic disease underlying the etiology of hyponatremia. We chose a low-dose treatment at the beginning which was adjusted according to the fluid-electrolyte balance. It would be advisable to start the treatment within the recovery regimen and under hospital conditions, since frequent monitoring of serum sodium levels is needed in the first hours after tolvaptan administration. In the first days of treatment, serum sodium levels, urinary output, and 24-h fluid balance should be closely evaluated. Based on previous experience indicating failure of fluid restriction, we permitted free access to liquids in all our three patients. Since too rapid sodium correction or fluctuations need to be avoided to prevent osmotic demyelination syndrome, withdrawal of other hyponatremia treatment options should be gradual. Tolvaptan should be started at a low dose of usually 3.75 mg and then slowly increased according to sodium values and urinary output. Serum sodium levels get prompt normalization or remain in low-normality range 24-48 hours after tolvaptan administration. In the first days of treatment, an increase in urinary output was observed as expected, but the volume progressively decreased in the following days and stabilized within 15-20 days although the patients remained polyuric.

To date, there are few data regarding the use of vaptans in paediatrics (11,12,13,14,15,16,17,18). The youngest patient treated was 2 years and 4 months old and the treatment dose ranged from 0.22 to 0.8 mg/kg (11). Tolvaptan has been effectively used in 28 patients with hyponatremia and heart failure, but none of the patients were affected by SIADH (12,13). Its use in the pediatric age group in patients with hypervolemic hyponatremia due to congestive cardiac failure with restrictive cardiomyopathy and in patients with massive edema due to nephrotic syndrome have been reported (14,15). There are reports of tolvaptan and conivaptan use in pediatric patients with acute hyponatremia who are resistant to isotonic or hypertonic fluids administration (16,17,18). Tolvaptan treatment was well-tolerated and considered to be a safe treatment in all the reported cases. Actually, EMA and FDA have approved tolvaptan only for the treatment of hyponatremia due to SIADH in adults (19,20,21). Conversely, conivaptan has previously been used in the management of hyperhydration during SIADH, but it is not yet licensed in Europe (16).

In conclusion, while further data are needed to strengthen its effectiveness and safety, we believe that tolvaptan can be a useful treatment option for euvolemic chronic hyponatremia due to SIADH in the pediatric age group. It is indeed noteworthy that through its oral administration, it can improve quality of life compared with intravenous administration of saline solution or fluid restriction. Finally, since increase in urine flow due to vaptans does not cause loss of electrolytes, no repletion is needed. For these reasons, vaptans utilization for the treatment of euvolemic and hypervolemic hyponatremias should actually be considered even in pediatric ages, especially when chronic diseases or syndromic conditions are responsible for disorders in the SIADH mechanism and in patients with severe hyponatremia symptoms.
